# Mapping Endangered Plant Distributions, Species Richness, and Climate Refugia Under SSP Climate Scenarios in South Korea

**DOI:** 10.3390/plants14243735

**Published:** 2025-12-08

**Authors:** Jae-Ho Lee, Eun-Seo Lee, Jae-Seok Lee, Chang-Wan Seo

**Affiliations:** 1 National Institute of Ecology, Seocheon 33657, Republic of Korea; jhlee14@nie.re.kr (J.-H.L.); pooky84@nie.re.kr (E.-S.L.); 2 Department of Biological Sciences, Konkuk University, Seoul 05029, Republic of Korea; jaeseok@konkuk.ac.kr

**Keywords:** threatened flora, habitat suitability, climate-change vulnerability, conservation prioritization, SSP2-4.5, SSP5-8.5, East Asia

## Abstract

Climate change significantly threatens the survival and distribution of endangered plants. This study quantified current and future patterns of habitat suitability and species richness for legally protected vascular plants in South Korea under two SSP climate scenarios. We modeled the distributions of 69 species designated as Class I or Class II endangered wild plants and evaluated in the Korean National Red List using IUCN criteria. Random Forest (RF) species distribution models were fitted with environmental predictors derived from 1 km^2^ national climate data for a 2010 baseline and SSP2-4.5 and SSP5-8.5 projections for the 2030s–2090s. Cross-validation indicated high model performance (mean AUC = 0.913; TSS = 0.818; Kappa = 0.605), and 61 species (88.4%) achieved an AUC ≥ 0.80. Current richness ranges from 0–40 species per municipality and peaks along the Baekdudaegan mountain range and on Jeju Island, whereas many lowland agricultural basins support few or no endangered plants. Under future climates, richness classes shift systematically: municipalities in low-richness classes expand, while high-richness classes contract, with stronger losses in southern lowlands and relative retention in northern and high-elevation areas, especially under SSP5-8.5. The resulting municipality- and national-park-level richness maps provide a quantitative basis for identifying potential climate refugia and prioritizing vulnerable regions and species.

## 1. Introduction

Global mean surface temperature has increased by approximately 1.1 °C since pre-industrial times (1850–1900), and this warming has already triggered rapid changes in the structure and functioning of marine, terrestrial, and freshwater ecosystems worldwide [1]. In parallel, the combined impacts of human activities and climate change have driven an unprecedented erosion of biodiversity, with up to one million species estimated to be at risk of extinction at the global scale [2]. One of the most visible manifestations of this crisis is the systematic shift of species’ geographic ranges along latitudinal and elevational gradients, with many taxa tracking warmer conditions by moving poleward and upward, often at accelerating rates consistent with increasing “climate velocities” [3,4,5]. To quantify such spatiotemporal dynamics and to support climate-informed conservation planning, species distribution models (SDMs), which statistically relate species occurrence data to environmental predictors, have become core tools for both ecological explanation and prediction [6,7]. Among the many SDM algorithms available, Random Forest (RF) has demonstrated particular strengths in conservation applications due to its ability to capture nonlinear responses and high-order interactions, its relatively robust performance with small and imbalanced samples, and its capacity to provide interpretable measures of variable importance [8,9,10]. As climatic inputs, high-resolution bioclimatic variables derived from downscaled regional climate models at ~1 km resolution have become essential for regional SDM studies, with recent applications increasingly utilizing nationally produced climate datasets that better capture local climatic conditions [11]. Future projections in this study were based on CMIP6 Shared Socioeconomic Pathways (SSPs), focusing on SSP2-4.5, an intermediate stabilization pathway, and SSP5-8.5, a fossil fuel–intensive high-emission pathway that brackets a wide range of potential future greenhouse gas trajectories [12].

The Korean Peninsula, and in particular the Republic of Korea (South Korea), harbors many legally protected endangered plant species with narrow geographic ranges whose persistence is likely to be strongly influenced by ongoing and future climatic change.

Building on recent advances in high-resolution climate datasets and species distribution models, the present study had four main objectives: (i) to estimate current (2010 baseline) habitat suitability and species richness patterns for 69 legally protected endangered vascular plant species in South Korea using Random Forest–based SDMs and 1 km bioclimatic variables; (ii) project changes in species distributions and richness under the intermediate SSP2-4.5 and high-emission SSP5-8.5 climate scenarios for the 2030s, 2050s, 2070s, and 2090s; (iii) quantify the relative contributions of six key temperature- and precipitation-related bioclimatic variables, with particular emphasis on annual mean temperature (BIO1) and precipitation of the driest month (BIO14); and (iv) identify municipalities and national parks that may serve as climate refugia or become highly exposed to richness losses. These analyses provide a quantitative basis for designing climate-adaptive conservation strategies and prioritizing management and restoration efforts for endangered flora in South Korea [6,7,12].

## 2. Materials and Methods

### 2.1. Study Area

**Figure 1:**
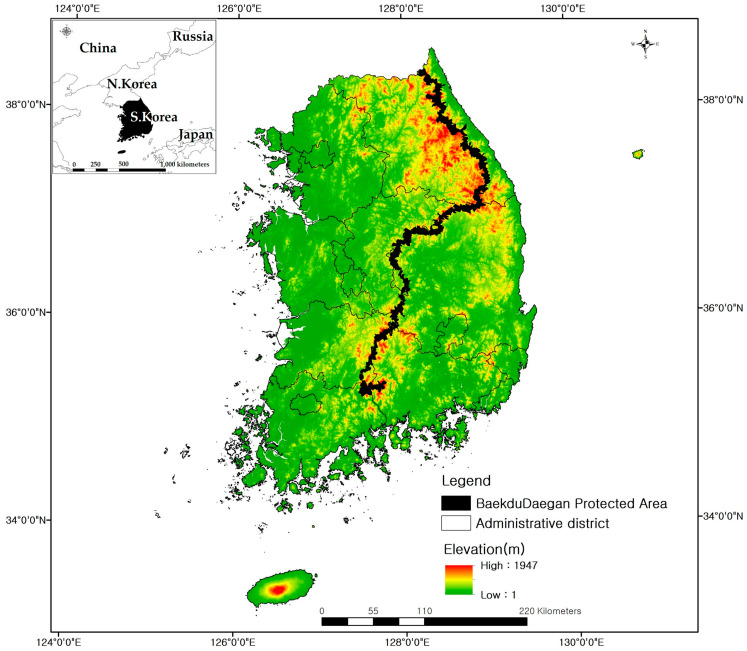
Study area in South Korea. The main panel shows the elevation (m) derived from a digital elevation model (DEM), with administrative boundaries and the Baekdudaegan Protected Area overlaid. The inset map indicates the location of South Korea within East Asia.

The study area encompasses the entire territory of the Republic of Korea (South Korea; approximately 33–38° N, 124–132° E), including both the mainland and adjacent islands (Figure 1). The total land area is approximately 100,033 km^2^, accounting for about half of the Korean Peninsula [13]. Around 70% of the country is mountainous, with mountains and highlands dominating the northern and eastern regions, while lowland plains and alluvial basins are mainly distributed in the west and south [13,14]. South Korea lies in the temperate monsoon climate zone of the mid-latitudes (approximately 33–38° N) and experiences hot, humid summers and cold, dry winters under the influence of the East Asian monsoon, with four distinct seasons [15]. Climatic zones can be broadly divided along latitudinal and elevational gradients into warm-temperate conditions in the south, temperate conditions in central regions, and cool-temperate conditions in the north and high mountain areas. Mean annual precipitation generally ranges from about 1000 to 1800 mm [13,14].

The natural vegetation is dominated by temperate deciduous broadleaf and coniferous forests, with warm-temperate evergreen broadleaf forests occurring along the southern coasts and on some islands, and subalpine–alpine coniferous forests occupying higher mountain zones. These patterns form clear latitudinal and elevational vegetation belts [14,15]. In particular, the Baekdudaegan mountain range, high mountain areas such as Mt. Hallasan, and insular regions in the South Sea and East Sea support high plant diversity due to their complex topography and heterogeneous microclimates, and they harbor numerous endemic and range-restricted species [14,15]. In this study, we defined the entire territory of South Korea, including these mountainous and insular ecosystems, as the spatial domain for assessing current and future habitat suitability and species richness patterns of 69 endangered plant species.

### 2.2. Species Occurrence Data

The target species of this study were vascular plants designated as endangered under the Wildlife Protection and Management Act of the Republic of Korea [16]. Species selection followed the 2022 revised endangered species list published by the Ministry of Environment and the Korean Red List and Red Data Books compiled by the National Institute of Biological Resources [16,17]. In total, 69 legally protected species were selected for which extinction risk is well documented and for which national-scale distribution data are available.

Occurrence records were compiled primarily from the National Institute of Ecology’s National Natural Environment Survey (1997–2021) and supplemented with published literature and national park monitoring data (2003–2022), all based on GPS-referenced locations. These data collectively cover a wide range of habitats across South Korea and provide the most comprehensive distribution information currently available for the target species.

To improve data quality and reduce spatial sampling bias, all occurrence records were reprojected to a 1 km × 1 km grid system. Duplicate records falling within the same grid cell were merged, and records judged to be erroneous or of low positional accuracy were removed. To avoid models based on single-cell occurrences, only species present in at least two independent grid cells were retained for analysis. This filtering procedure helped minimize spatial autocorrelation and ensured that the remaining records were suitable for modeling at the 1 km spatial resolution used in this study.

All 69 target species are legally designated as Class I or Class II endangered wild plants under the Wildlife Protection and Management Act of the Republic of Korea, and each species has been evaluated in the Korean National Red List using IUCN criteria, with categories spanning Regionally Extinct (RE), Critically Endangered (CR), Endangered (EN), Vulnerable (VU), and Near Threatened (NT). The national legal classes and Red List categories for all target species are provided in Table S1.

### 2.3. Environmental Variables

Environmental predictors were derived from high-resolution climate data provided by the Korea Meteorological Administration (KMA) at 1 km^2^ spatial resolution [18]. These data were generated through statistical ensemble averaging of five regional climate models (HadGEM3-RA, WRF, CCLM, GRIMs, RegCM4), providing improved representation of local climatic conditions across the Korean Peninsula. Six bioclimatic variables were selected as environmental predictors: annual mean temperature (BIO1), mean diurnal range (BIO2), isothermality (BIO3), annual precipitation (BIO12), precipitation of the wettest month (BIO13), and precipitation of the driest month (BIO14) (Table 1). These variables are widely recognized as key climatic determinants of species distributions and have been extensively applied in species distribution modeling studies [6]. All environmental layers were standardized to a common coordinate system (WGS 1984) and resampled to a consistent spatial resolution of 1 km^2^. Current climate conditions were represented by the climatological normal period of 1995–2014, which we refer to as the 2010 baseline throughout this study. To address potential multicollinearity among predictors, we calculated pairwise Pearson correlation coefficients for all variable combinations. When correlation coefficients exceeded 0.7, we retained variables based on ecological relevance and their importance in previous distribution modeling studies, following established protocols for collinearity reduction [19]. The final set of six bioclimatic variables exhibited acceptable levels of intercorrelation and was deemed suitable for inclusion in the Random Forest modeling framework.

### 2.4. Future Climate Scenarios

To project changes in species distributions under future climate conditions, we used the Shared Socioeconomic Pathways (SSPs) adopted in the IPCC Sixth Assessment Report [12]. Two scenarios were selected: SSP2-4.5, representing an intermediate greenhouse gas emission pathway with continued but moderate climate mitigation efforts, and SSP5-8.5, representing a high-emission pathway characterized by sustained reliance on fossil fuels. Future climate data for each scenario were obtained from the Korea Meteorological Administration (KMA), which provides statistically ensembled climate projections at 1 km^2^ spatial resolution based on five regional climate models under the CMIP6 framework (Korea Meteorological Administration, 2024). The ensemble approach integrates outputs from multiple regional climate models to reduce uncertainties inherent in individual model projections [18]. Projections were generated for four future time periods: the 2030s (2021–2040), 2050s (2041–2060), 2070s (2061–2080), and 2090s (2081–2100).

### 2.5. Species Distribution Modeling

Species distribution modeling was conducted using the Random Forest (RF) algorithm [8,20]. RF is well suited to capturing complex and nonlinear relationships between species occurrences and environmental variables, and can effectively handle high-order interactions among predictors [9,21]. It is also relatively robust to overfitting and has been shown to perform well for rare and endangered species with limited sample sizes [10,22]. All models were implemented in R (version 4.3.0; R Foundation for Statistical Computing, Vienna, Austria) using the randomForest package. For each species, we generated pseudo-absence points equal in number to the observed presences. Pseudo-absences were randomly sampled across the entire study area without additional spatial constraints, in order to maximize environmental contrast between presence and absence locations. Model parameters were set to n_tree_ = 500 (number of trees) and m_try_ = 2 (number of predictor variables randomly selected at each split). To evaluate model performance, we applied 5-fold cross-validation. In each run, 80% of the data were used for model training and the remaining 20% for testing. This procedure was repeated 10 times for each species, with pseudo-absences resampled independently in every repetition. Final model predictions and evaluation metrics were summarized as mean values across the 10 repetitions.

### 2.6. Model Evaluation

The predictive performance of the Random Forest models was evaluated using the repeated 5-fold cross-validation procedure described above. For each species, and for each repetition and fold, model performance was calculated on the held-out test data and then averaged across folds and repetitions to obtain a final evaluation value per species. As the primary, threshold-independent metric, we used the area under the receiver operating characteristic curve (AUC) [23]. AUC values range from 0 to 1, with 0.5 indicating performance equivalent to random classification and values closer to 1 indicating better discrimination between presence and absence. In this study, we refer to the area under the ROC curve simply as AUC. In addition to AUC, we used two threshold-dependent metrics—True Skill Statistic (TSS) and Cohen’s Kappa—as supplementary measures of model performance [24]. Both TSS and Kappa are derived from the confusion matrix and jointly account for correct and incorrect classifications of presence and absence. Their values range from −1 to 1, with values ≤ 0 indicating equal or worse performance than random expectation and values closer to 1 indicating higher predictive accuracy. For all three metrics (AUC, TSS, and Kappa), we used the mean values obtained from the test data across the 10 repetitions as the final performance indices in species-level and across-species analyses.

### 2.7. Variable Importance Analysis

To assess the relative contribution of each environmental variable to the species distribution predictions, we calculated variable importance using the permutation-based approach implemented in the Random Forest algorithm [8,9]. This method measures the decrease in model accuracy (Mean Decrease in Accuracy, MDA) when a specific variable is randomly shuffled, thus disrupting the relationship between that variable and species occurrence. A larger decrease in accuracy indicates that the variable plays a more significant role in explaining the species’ distribution. For each species, the importance values of the six bioclimatic variables (BIO1, BIO2, BIO3, BIO12, BIO13, BIO14) were extracted from the Random Forest models. These values were then normalized within each species, with the importance scores summed to 1 for easier comparison. Based on these normalized scores, we ranked the variables from 1 (most important) to 6 (least important) for each species. In addition to the individual species-level rankings, we summarized the occurrence frequencies of the top-ranked variables (those ranked 1st), calculated the mean ranks for each variable across species, and identified the most common variable combinations appearing among the top three ranks. This analysis allowed us to identify the primary environmental factors influencing species distribution on an individual level, as well as identify shared climatic gradients and variable combination patterns across the 69 species.

### 2.8. Species Richness Analysis

Species richness was defined as the number of species in each grid cell whose predicted climatic suitability exceeded a species-specific threshold. For each species, the optimal threshold was determined as the value that maximized the True Skill Statistic (TSS), i.e., the difference between the true positive rate and the false positive rate, thereby providing the best balance between omission and commission errors [24]. Predicted suitability values were obtained from the Random Forest models as occurrence probabilities for each 1 km × 1 km grid cell. For subsequent analyses, species richness was summarized at two spatial units: (i) municipalities (Si/Gun/Gu administrative units) and (ii) national parks. At each spatial unit, we counted the number of species for which at least one grid cell within the unit exceeded the species-specific TSS-based threshold, and we also calculated the mean richness per unit based on grid-level counts. This procedure was applied to the current (2010 baseline) climate as well as to future climate scenarios (SSP2-4.5 and SSP5-8.5) for the four projection periods. The resulting municipality- and national park–level richness patterns provide a spatially aggregated view of current and future species richness, enabling the identification of high-biodiversity hotspots, areas of projected richness decline or increase, and contrasts in climate-driven change between administrative and protected areas.

## 3. Results

### 3.1. Model Performance Evaluation

**Figure 2:**
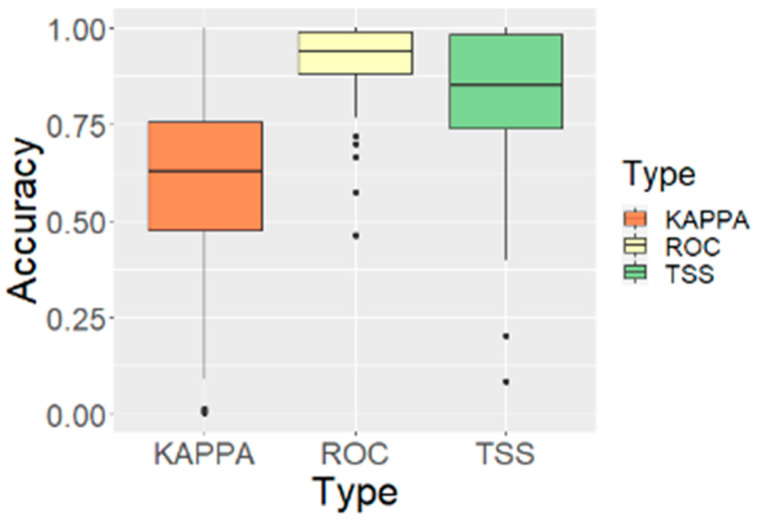
Distribution of model accuracies (AUC) across 69 endangered plant species in Korea.

Across the 69 endangered plant species modeled with Random Forest, the mean AUC was 0.913 ± 0.103, while the mean TSS and Kappa were 0.818 ± 0.187 and 0.605 ± 0.232, respectively (Table 2). AUC values ranged from 0.462 to 1.000, TSS from 0.085 to 1.000, and Kappa from 0.004 to 1.000. In this study, the area under the receiver operating characteristic curve (AUC) was used as the primary, threshold-independent performance metric, with TSS and Kappa reported as complementary, threshold-dependent indices (Figure 2).

**Figure 3:**
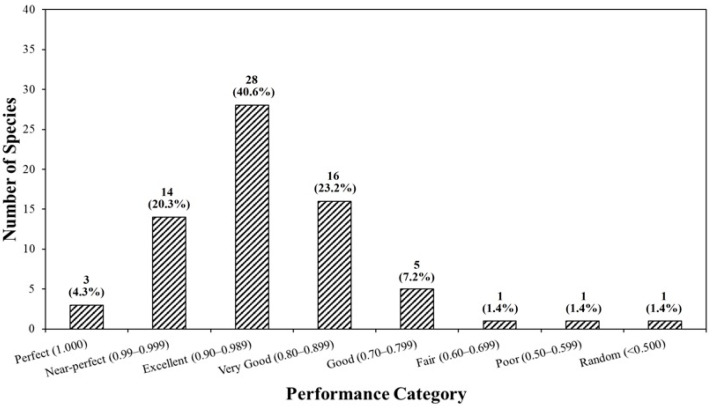
Number of species by performance category.

When species were grouped by AUC performance classes, 3 species (4.3%) fell into the Perfect class (AUC = 1.000), 14 species (20.3%) into Near-perfect (0.990–0.999), 28 species (40.6%) into Excellent (0.900–0.989), and 16 species (23.2%) into Very Good (0.800–0.899). Five species (7.2%) were classified as Good (0.700–0.799), and only one species each (1.4% per class) fell into the Fair (0.600–0.699), Poor (0.500–0.599), and Random (< 0.500) categories (Table 3, Figure 3). Overall, 61 out of 69 species (88.4%) had an AUC ≥ 0.800. The three species with an AUC = 1.000 were *Scrophularia takesimensis*, *Bupleurum latissimum*, and *Diapensia lapponica*. The lowest AUC was observed for *Viola raddeana* (0.462), and relatively low AUC values were also obtained for *Arctous rubra* (0.573) and *Cypripedium guttatum* (0.665). Most remaining species showed AUC values above 0.80. Species-specific model performance metrics, primary climatic drivers, and variable combination patterns for all 69 species are provided in Table S2.

### 3.2. Environmental Variable Importance

**Figure 4:**
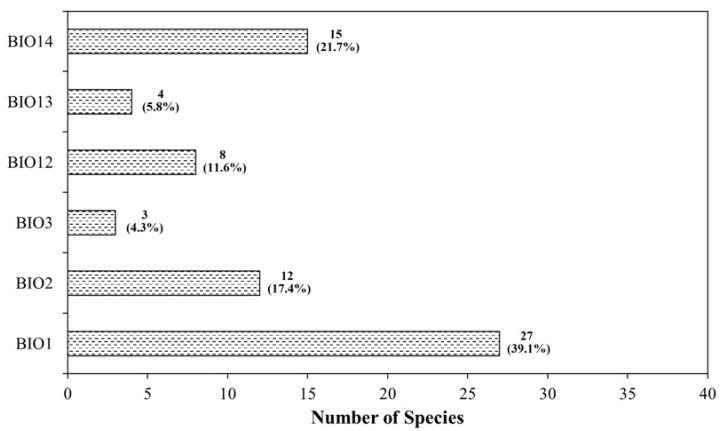
Number (and percentage) of species for which each bioclimatic variable was ranked as the most important (RF permutation importance).

The permutation importance analysis of six bioclimatic variables revealed that annual mean temperature (BIO1) was the most important variable for 27 species (39.1%) out of the 69 species studied (Figure 4). Precipitation of the driest month (BIO14) ranked as the most important for 15 species (21.7%), while mean diurnal range (BIO2) was the most important for 12 species (17.4%), and annual precipitation (BIO12) for 8 species (11.6%). Precipitation of the wettest month (BIO13) ranked as the most important for 4 species (5.8%), and isothermality (BIO3) was the most important for 3 species (4.3%). When grouping the most important variables, temperature-related variables (BIO1–BIO3) ranked first for 42 species (60.9%), while precipitation-related variables (BIO12–BIO14) ranked first for 27 species (39.1%). Based on the mean rank (1 = most important, 6 = least important), BIO14 had the lowest average rank of 2.97, followed by BIO1 and BIO2, with average ranks of 3.07 and 3.14, respectively. BIO12 had a mean rank of 3.67, while BIO3 and BIO13 had relatively higher average ranks of 4.06 and 4.09, respectively. The frequency of inclusion of variables in the top 3 ranks was high for BIO1, BIO14, and BIO2, while BIO3 and BIO13 were more commonly ranked 4th to 6th (Table 4).

### 3.3. Heterogeneity in Species-Specific Variable Importance Rankings

**Figure 5:**
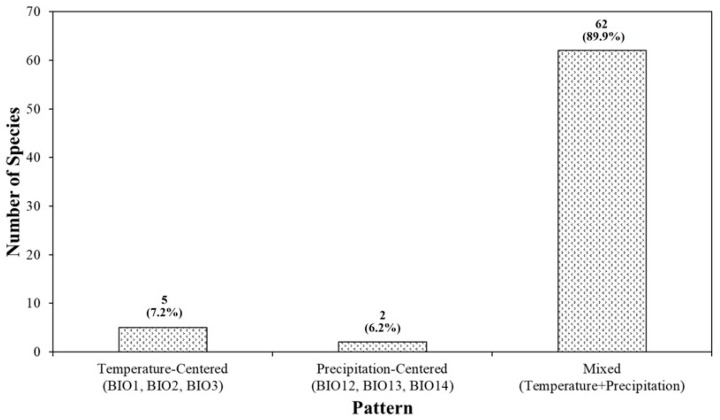
Counts of species by top-three variable-combination pattern: temperature-centered (BIO1–BIO3), precipitation-centered (BIO12–BIO14), and mixed.

Comparison of 1st–6th-rank variable combinations showed marked among-species variation in climatic response patterns. For *Euryale ferox*, annual mean temperature (BIO1) was ranked first, with annual and wettest-month precipitation (BIO12, BIO13) also appearing among the highest ranks, forming a combination in which both temperature and precipitation variables are important. For *Nymphaea tetragona*, precipitation of the driest month (BIO14) ranked first, followed by isothermality (BIO3) and mean diurnal range (BIO2), yielding a configuration in which dry-season moisture and thermal variability occupied the top ranks. When species were classified according to the composition of their top three permutation-importance variables, three patterns were identified. The Temperature-centered type, in which all top-three variables were temperature-related (BIO1–BIO3), included 5 species (7.2%). The Precipitation-centered type, in which all top-three variables were precipitation-related (BIO12–BIO14), included 2 species (2.9%). The remaining 62 species (89.9%) were assigned to the Mixed type, in which the top three variables contained at least one temperature variable and at least one precipitation variable (Table 5, Figure 5). The Temperature-centered group included, among others, *Viola mirabilis*, *Cicuta virosa*, *Menyanthes trifoliata*, and *Arctous rubra*. The Precipitation-centered group consisted of *Thrixspermum japonicum* and *Diapensia lapponica*. Species assigned to the Mixed type showed various combinations of temperature and precipitation variables—most frequently involving BIO1, BIO2, BIO14, and BIO12—within their top three ranks. Mean AUC values by pattern were 0.839 for Temperature-centered species, close to 1.000 for Precipitation-centered species, and 0.914 for Mixed species, with minimum and maximum AUC values differing among patterns and Mixed species showing the widest performance range.

### 3.4. Current Species Distributions and Species Richness

**Figure 6:**
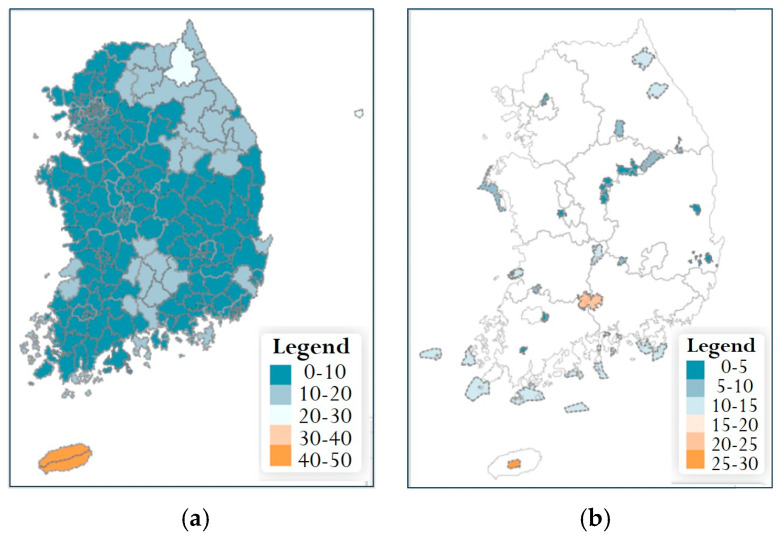
Baseline species richness of endangered plants in South Korea (2010). Spatial distribution of species richness for 69 endangered plant species under current climate conditions (2010): (**a**) species richness at the municipality level (Si/Gun/Gu administrative units); (**b**) species richness at the national park level.

Under current climate conditions (2010 baseline), municipality-level species richness for the 69 endangered plant species showed that most Si/Gun/Gu administrative units fell within the 10–20 species class (Figure 6a). Municipalities in the lowest richness class (0–10 species) were mainly located in the northern part of the country and in some inland areas of the eastern region, whereas municipalities with 20–30 or more species were limited to relatively few areas. Jeju Island was classified in the 30–40 species class, indicating higher richness than most other regions. At the national park scale, most parks belonged to the 0–5 or 6–10 species classes (Figure 6b). A few national parks in the central inland region and on Jeju Island fell into the 15–20 species class, recording relatively high numbers of endangered plant species compared with other parks. No national parks were classified in richness classes exceeding 20 species (i.e., 20–25 or 25–30 species), as shown in Figure 6b.

### 3.5. Changes in Species Richness Under Future Climate Scenarios

**Figure 7:**
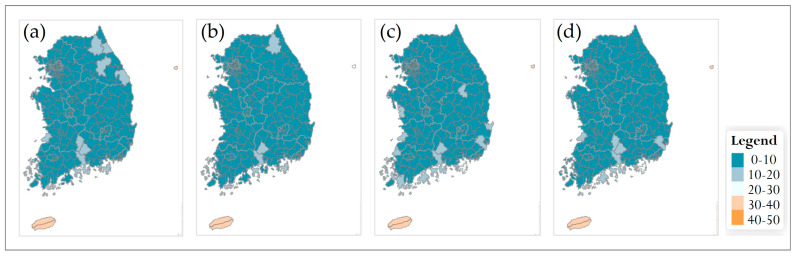
Future species richness of endangered plants at the municipality level in South Korea under the SSP2-4.5 scenario for four time periods: (**a**) 2030s, (**b**) 2050s, (**c**) 2070s, and (**d**) 2090s. A common legend for all panels is shown on the right.

**Figure 8:**
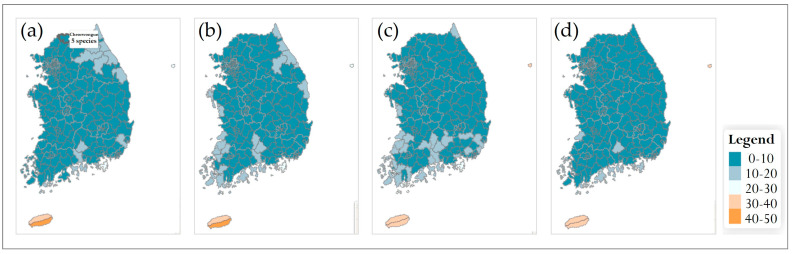
Future species richness of endangered plants at the municipality level in South Korea under the SSP5-8.5 scenario for four time periods: (**a**) 2030s, (**b**) 2050s, (**c**) 2070s, and (**d**) 2090s. A common legend for all panels is shown on the right.

**Figure 9:**
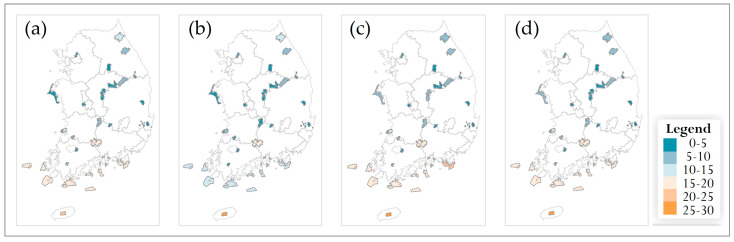
Future species richness of endangered plants in national parks of South Korea under the SSP2-4.5 scenario for four time periods: (**a**) 2030s, (**b**) 2050s, (**c**) 2070s, and (**d**) 2090s. A common legend for all panels is shown on the right.

**Figure 10:**
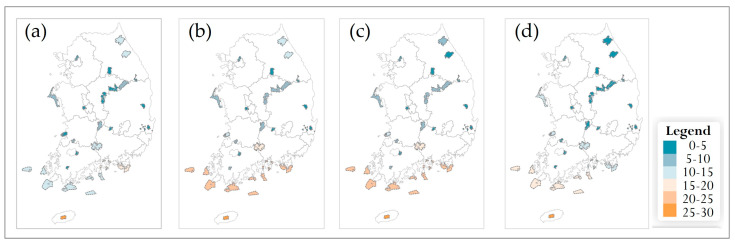
Future species richness of endangered plants in national parks of South Korea under the SSP5-8.5 scenario for four time periods: (**a**) 2030s, (**b**) 2050s, (**c**) 2070s, and (**d**) 2090s. A common legend for all panels is shown on the right.

Under future climate conditions, species richness of the 69 endangered plant species was projected for two climate scenarios (SSP2-4.5 and SSP5-8.5) and four future time periods (2030s, 2050s, 2070s, 2090s). Municipality-level (Si/Gun/Gu) patterns under each scenario are shown in Figure 7 and Figure 8, and results for national parks are presented in Figure 9 and Figure 10. For both spatial units, species richness was classified into six classes (0–5, 5–10, 10–15, 15–20, 20–25, and 25–30 species) to facilitate comparison of spatial and temporal changes.

#### 3.5.1. Changes in Species Richness at Municipality Level

In the SSP2-4.5 scenario, municipality-level richness patterns remained broadly similar across the 2030s–2090s (Figure 7). Throughout all time periods, most municipalities belonged to the intermediate richness classes of 5–10 and 10–15 species. In the 2030s, municipalities in the lowest richness class (0–5 species) were scattered mainly across the northern region and parts of the inland east, whereas municipalities with ≥15 species were restricted to a small number of areas. This overall structure changed only gradually toward the 2050s and 2070s, with a slight increase in 0–5-species municipalities in some central and southern inland or coastal areas. By the 2090s, a limited number of southern and southern coastal municipalities had shifted to the 0–5-species class, but the 5–10-species class still dominated at the national scale. Jeju Island consistently maintained relatively high richness, remaining in the ≥20-species classes across all time periods.

In the SSP5-8.5 scenario, municipality-level patterns were qualitatively similar but showed more pronounced temporal changes (Figure 8). In the 2030s, the 5–10-species class was again widespread nationwide, with only a few northern and coastal municipalities falling into the 0–5-species class. However, from the 2050s onward, an increasing number of municipalities in the southern and western coastal regions, as well as some inland areas, shifted to the lowest richness class, while the extent of the 10–15-species class gradually contracted. By the 2090s, most municipalities were classified in the 5–10-species class, and only a few retained ≥15 species. As under SSP2-4.5, Jeju Island remained one of the richest areas, with municipalities persistently falling into the 20–25- or 25–30-species classes throughout the century.

#### 3.5.2. Changes in Species Richness Within National Parks

At the national park scale, richness patterns were relatively stable under SSP2-4.5 (Figure 9). In the 2030s and 2050s, many high-mountain and montane parks maintained intermediate to high richness classes. In particular, Mt. Seoraksan, Mt. Odaesan, Mt. Sobaeksan, and Mt. Deogyusan National Parks consistently contained ≥10 endangered plant species. During the 2070s and 2090s, these high-elevation parks continued to show comparatively high richness, while some low-elevation parks exhibited slight declines and shifted to lower richness classes. Overall, the distribution of richness classes changed only modestly over time under SSP2-4.5.

In contrast, temporal changes in national park richness were more pronounced under SSP5-8.5 (Figure 10). Although high-elevation parks initially retained relatively high richness in the 2030s and 2050s, many lowland and coastal parks showed progressive declines from the 2050s onward. By the 2070s and 2090s, a larger proportion of parks in lowland plains and coastal regions fell into the 0–5- or 5–10-species classes, and even some high-mountain parks shifted to lower richness categories. Across both scenarios, high-elevation and montane parks generally supported higher richness than lowland and coastal parks, but decreases in richness were clearly more marked under SSP5-8.5 than under SSP2-4.5.

#### 3.5.3. Temporal Patterns and General Trends

Across both climate scenarios and spatial scales, future richness patterns diverged progressively from the 2010 baseline. In the 2030s, most municipalities and national parks remained in the same or adjacent richness classes as at present, and differences between SSP2-4.5 and SSP5-8.5 were minor (Figure 7, Figure 8, Figure 9 and Figure 10). From the 2050s onward, however, the spatial extent of low-richness classes (0–5 species) increased, particularly in southern and western coastal municipalities and in low-elevation parks, whereas the extent of medium- to high-richness classes (10–15 and ≥15 species) decreased. These shifts were evident under SSP2-4.5 but became more pronounced under SSP5-8.5, where many municipalities and several parks transitioned from medium- to low-richness classes by the 2090s. Overall, the projections indicate an intensification of latitudinal and elevational gradients in endangered-plant richness, with relatively species-rich, high-elevation regions in the central and northern parts of the country contrasted against increasingly species-poor, low-elevation municipalities and parks in the south. This emerging spatial polarization highlights the importance of mountainous municipalities and national parks as potential climate refugia and underscores the need for targeted management actions in southern lowland regions where richness declines are projected to be most severe.

## 4. Discussion

### 4.1. Model Performance and Methodological Strengths

Random Forest models developed in this study showed generally high predictive performance for the 69 endangered plant species in South Korea. Cross-validation yielded a mean AUC of 0.913 ± 0.103, with mean TSS and Kappa values of 0.818 ± 0.187 and 0.605 ± 0.232, respectively, and the AUC distribution was clearly skewed toward the upper performance classes. In total, 61 species (88.4%) had an AUC ≥ 0.80, and three species—*Scrophularia takesimensis*, *Bupleurum latissimum*, and *Diapensia lapponica*—showed perfect discrimination with an AUC = 1.000. These results are consistent with previous studies reporting that RF can provide robust predictions even for rare and endangered species with limited occurrence data [9,10,21,25]. The strong performance observed here is closely linked to the algorithmic properties of RF, including its ability to learn nonlinear species–environment relationships, =internally accommodate multiple predictors and their interactions, and reduce overfitting through bootstrap aggregation and ensemble averaging [8,9]. In contrast, several species such as *Viola raddeana*, *Arctous rubra*, and *Cypripedium guttatum* showed relatively low performance (AUC < 0.7), indicating that their distribution patterns are not fully captured by bioclimatic variables alone. For these species, unmodeled factors such as soil conditions, microclimate, disturbance regimes, or biotic interactions may play comparatively stronger roles, highlighting that SDMs are constrained by the environmental gradients represented in the predictor set [6].

### 4.2. Environmental Drivers of Species Distributions

The environmental variable importance analysis showed that annual mean temperature (BIO1) was the most important predictor for 27 of the 69 species (39.1%), followed by precipitation of the driest month (BIO14) for 15 species (21.7%). Mean diurnal range (BIO2) was ranked first for 12 species (17.4%), annual precipitation (BIO12) for 8 species (11.6%), precipitation of the wettest month (BIO13) for 4 species (5.8%), and isothermality (BIO3) for 3 species (4.3%). Based on mean rank (1 = most important, 6 = least important), BIO14, BIO1, and BIO2 had relatively low (i.e., more important) average ranks, whereas BIO3 and BIO13 tended to have higher mean rank values. These patterns are consistent with biogeographical evidence from temperate East Asia indicating that thermal conditions constrain the broad-scale limits of plant distributions, with dry-season water availability acting as an additional limiting factor [26,27,28,29]. When the top three variables were grouped into patterns, only 5 species (7.2%) were classified as Temperature-centered, with all top-three variables belonging to the temperature group (BIO1–BIO3), and 2 species (2.9%) as Precipitation-centered, with all top-three variables belonging to the precipitation group (BIO12–BIO14). The remaining 62 species (89.9%) were assigned to the Mixed type, in which both temperature- and precipitation-related variables appeared within the top three positions. For many species, annual mean temperature, diurnal temperature range, and dry-season or annual precipitation formed diverse combinations of important predictors, which aligns with the general view from SDM studies that species distributions are often shaped by multiple environmental gradients rather than a single climatic axis [7,30]. At the opposite end of the ranking, the lowest-importance (6th-rank) variable was most frequently BIO3 or BIO2. Although isothermality (BIO3) and mean diurnal range (BIO2) were top-ranked for some species, they were more often assigned to lower ranks, indicating that fine-scale temperature variability was evaluated as less important than coarser thermal and moisture gradients (e.g., BIO1, BIO14) for many of the endangered plant species at the spatial resolution used in this study.

Similar patterns, with temperature and dry-season moisture emerging as dominant constraints on plant habitat suitability, have been reported in recent SDM applications to climate-sensitive indicator plants and single tree species in South Korea under climate change scenarios [31,32,33].

### 4.3. Spatial Structure of Current Distributions

Under current (2010 baseline) climate conditions, the predicted species richness of the 69 endangered plant species showed a pronounced spatial structure. At the municipality (Si/Gun/Gu) level, relatively high richness values were distributed broadly along the Baekdudaegan mountain axis, with a core band extending through central-eastern Gangwon-do, northern Gyeongsangbuk-do, and northeastern Chungcheongbuk-do. Most municipalities fell within the 10–20 species class, but some areas adjacent to this mountain axis reached richness levels exceeding 20 species. In contrast, lowland municipalities along the western and southern coasts and the major interior plains were dominated by the 0–10 species class, indicating comparatively low richness of endangered plants. Jeju Island was classified in a higher richness class (e.g., 30–40 species) than most other regions, displaying a distinct pattern compared with the southern coastal and lowland areas.

At the national park scale, mountain parks located along the Baekdudaegan, such as Mt. Seoraksan, Mt. Odaesan, Mt. Sobaeksan, and Mt. Deogyusan, maintained relatively high numbers of endangered plant species. Some island and coastal parks also formed localized high-richness patches, driven by a small number of endemic or range-restricted species. In contrast, low-elevation and lowland-type parks were generally assigned to lower richness classes. Overall, these patterns are broadly consistent with previous findings that complex topography and microclimatic gradients in mountain and island ecosystems promote the concentration of endemic and range-restricted species [27,34,35].

### 4.4. Future Range Shifts Under Climate Scenarios

Under the two future climate scenarios (SSP2-4.5 and SSP5-8.5), projected changes in species richness for the 69 endangered plant species showed a generally time-progressive pattern in both cases. In SSP2-4.5, the main axis of high suitability broadly persisted through the 2030s and 2050s, with some aggregation of high-suitability areas in high-elevation zones. By the 2070s and 2090s, the area of high suitability expanded or concentrated in parts of the high mountains, while low-elevation regions showed an increase in low-richness classes, indicating a gradual contraction of suitable conditions in the lowlands. Under this moderate-emission pathway, climate change influenced the distributions of endangered plants, but high-elevation areas behaved relatively more stably and tended to retain higher richness levels over time. In contrast, SSP5-8.5 assumed more rapid and intensive climate warming, and pronounced changes in species richness emerged earlier in high-elevation regions, already by the 2030s. From the 2050s onward, high-suitability areas in the mountains began to shrink, while richness in lowland regions declined rapidly. By the 2070s and 2090s, range shifts toward higher latitudes and elevations became more evident, reflecting a progressive displacement of suitable habitat northwards and upslope. Under this scenario, habitat loss risks were relatively greater for species adapted to high mountains and extreme environments, and fragmentation of suitable habitat proceeded more rapidly with continued warming.

These patterns are consistent with studies showing that accelerating poleward and upslope shifts of isotherms can rapidly alter the spatial position of climatic zones and thereby reshape the boundaries between lowland and montane environments [3,4,5]. In mountain regions, climate-driven habitat loss interacts with strong topographic constraints, amplifying the risk of range contraction and local extinction as available high-elevation area diminishes [36,37,38]. Our finding that montane and high-latitude areas tend to retain higher richness for longer under moderate emission scenarios is consistent with recent vulnerability assessments of alpine plant species in other mountain regions, such as the Tibetan–Himalayan hotspot [39], and with global analyses emphasizing the role of mountain systems as potential climate refugia.

### 4.5. Differential Vulnerability Among Administrative and Protected Areas

Species richness patterns analyzed at the municipality (Si/Gun/Gu) and national park scales indicate that exposure and response capacity to climate change differ among management units. Under current climate conditions, mountainous municipalities along the Baekdudaegan in eastern Gangwon-do, Gyeongsangbuk-do, and Chungcheongbuk-do, together with high-elevation national parks such as Mt. Seoraksan, Mt. Odaesan, Mt. Sobaeksan, and Mt. Deogyusan, maintain high levels of endangered plant richness. Under the SSP2-4.5 scenario, these areas tended to retain comparatively high richness over the long-term. Several municipalities on Jeju Island and Mt. Hallasan National Park also fell into higher richness classes than surrounding lowland regions, suggesting that they are likely to remain important concentration areas for endangered plants in the future.

In contrast, lowland municipalities along the western and southern coasts, extensive interior plains, and low-elevation or lowland-type national parks already show low current richness and exhibit increased frequency of transitions into the 0–5 species class over time under SSP5-8.5, indicating relatively high vulnerability. This spatial asymmetry shows that the same regional climate change signal can manifest differently across management units, and that administrative areas and protected areas may need differentiated roles and strategies. High-latitude and high-elevation municipalities, together with northern and high-mountain national parks, can function as potential climate refugia and primary recipient areas for newly suitable habitats for endangered plants, and are therefore priority targets for proactive conservation actions such as maintaining habitat quality and facilitating establishment and spread [40,41]. In contrast, in southern lowland parks and low-richness municipalities complementary strategies such as habitat restoration linked to land-use planning, strengthening landscape connectivity, and ex situ conservation or reintroduction are likely to become increasingly important [42].

Differences in richness patterns between the two scenarios also tended to widen over time, with greater losses in lowland and low-elevation management units in the later periods (2070s–2090s) under SSP5-8.5. This indicates that the spatial configuration of endangered plant assemblages and the conservation burden borne by each management unit can change substantially depending on the emission pathway, in line with previous assessments that global greenhouse gas mitigation reduces long-term conservation costs and risks [43,44].

### 4.6. Implications for Conservation Prioritization

The current and future species richness patterns derived in this study can be used to refine spatial conservation priorities for endangered plants. Municipalities and national parks that consistently maintain high richness across the baseline (2010) and both climate scenarios (SSP2-4.5 and SSP5-8.5) can be regarded as candidate climate refugia. In such areas, long-term conservation and management plans that minimize land-use change and additional disturbances are required, given their sustained potential to support multiple endangered species [45,46,47]. Conversely, areas that currently exhibit low richness but are projected to gain species or develop new patches of high suitability under future scenarios represent promising targets for proactive conservation and restoration efforts [47]. Species-specific model performance and information on key environmental drivers can also support differentiated conservation strategies. Species with high AUC values and clear responses to major climatic gradients (e.g., BIO1, BIO14) are suitable for using SDM projections to identify candidate areas for habitat expansion, corridors for enhancing connectivity, and other spatial priorities under climate change. In contrast, species with low performance or strong apparent sensitivity to a single predictor likely require additional investigation of non-climatic factors such as soil properties, topography, disturbance regimes, and interactions with native or invasive species [48]. For these species, population-level interventions—such as translocation, ex situ propagation, and fine-scale microhabitat management—may need to accompany broader climate and land-use policies. By combining SDM outputs with climate scenario analyses, it becomes possible to distinguish areas that function as climate refugia, potential new suitable habitats, and regions of high risk of richness decline, and to assign stepwise and region-specific conservation priorities accordingly [45,46,47]. These spatially explicit results can be integrated into future plans for expanding protected areas, designing restoration projects, and coordinating land-use planning at national and local levels.

## 5. Conclusions

This study used Random Forest-based species distribution models to quantitatively assess current (2010 baseline) habitat suitability and species richness patterns, as well as future changes under two climate scenarios (SSP2-4.5 and SSP5-8.5), for 69 endangered plant species in South Korea. Model performance was generally high, with most species showing an AUC ≥ 0.80, and the resulting projections revealed marked spatial heterogeneity in endangered plant distributions, including high-richness areas concentrated along the Baekdudaegan mountain range and on Jeju Island. Variable importance analysis identified annual mean temperature (BIO1) and precipitation of the driest month (BIO14) as key environmental drivers, and for many species, mixed combinations of temperature- and precipitation-related variables formed the dominant importance patterns. Future projections indicate that in both scenarios, species richness in lowland and low-elevation areas tends to decline over time, while richness becomes relatively concentrated in central–northern mountain regions and certain island areas. Under SSP2-4.5, the current high-richness axis is relatively well maintained through the mid-century, and the magnitude and rate of change remain moderate, whereas under SSP5-8.5, lowland contraction, upslope and poleward shifts, and habitat fragmentation become more pronounced in the later periods (2070s–2090s). These spatiotemporal patterns suggest that the Baekdudaegan and high-mountain and island regions are likely to function as major candidates for climate refugia and newly suitable habitats, whereas habitat restoration and enhancement of landscape connectivity emerge as priority tasks in lowland, coastal, and plain regions. The spatially explicit results at the municipality and national park levels provide directly usable baseline information for protected area management, local-scale conservation priority setting, and integration into land-use planning.

At the same time, the models in this study are primarily based on coarse-scale bioclimatic variables and therefore do not fully account for non-climatic factors such as soil properties, microclimate, dispersal limitations, biotic interactions, and anthropogenic disturbances. Future work should aim to increase predictive realism and management relevance by incorporating high-resolution microclimate and soil datasets, explicitly modeling landscape connectivity and species-specific dispersal characteristics, and integrating biotic interactions and disturbance regimes into the modeling framework.

## Figures and Tables

**Table 1:** Summary of bioclimatic variables used in the study, including their codes, descriptions, units, sources, and spatial resolution.

Variable Code	Description	Unit	Source	Resolution
BIO1	Annual mean temperature	°C	KMA	1 km
BIO2	Mean diurnal temperature range	°C	KMA	1 km
BIO3	Isothermality (BIO2/BIO7 × 100)	-	KMA	1 km
BIO12	Annual precipitation	mm	KMA	1 km
BIO13	Precipitation of wettest month	mm	KMA	1 km
BIO14	Precipitation of driest month	mm	KMA	1 km

**Table 2:** Overall average performance metrics of the random forest model.

Performance Metric	Mean	Min	Max	Standard Deviation
AUC	0.913	0.462	1.000	0.103
TSS	0.818	0.085	1.000	0.187
Kappa	0.605	0.004	1.000	0.232

Note: The metrics were calculated based on a 5-fold cross-validation approach across the 69 endangered plant species. AUC (Area Under the ROC Curve) is a threshold-independent measure summarizing the model’s discriminative ability. TSS (True Skill Statistic) and Kappa are threshold-dependent auxiliary metrics indicating the model’s overall agreement and predictive power beyond chance.

**Table 3:** Distribution of the 69 Endangered Plant Species by Individual Model Performance Class.

Performance Class	AUC Range	Number of Species	Percentage (%)
Perfect	1.000	3	4.3
Near-perfect	0.990–0.999	14	20.3
Excellent	0.900–0.989	28	40.6
Very Good	0.800–0.899	16	23.2
Good	0.700–0.799	5	7.2
Fair	0.600–0.699	1	1.4
Poor	0.500–0.599	1	1.4
Random	<0.500	1	1.4
Total		69	100

Note: The performance class thresholds follow the general guidelines for evaluating species distribution models. A large majority of the models (>88%) achieved an AUC score of 0.800 or above (Very Good to Perfect), demonstrating strong predictive power across the majority of the study species. Percentages may not sum to exactly 100% because of rounding.

**Table 4:** Summary of Relative Importance of Bioclimatic Variables in Predicting Endangered Plant Species Distributions.

Code	Variable	Species with 1st-Rank Importance (n)	Mean Rank †
BIO1	Annual mean temperature	27	3.07
BIO2	Mean diurnal range	12	3.14
BIO3	Isothermality	3	4.06
BIO12	Annual precipitation	8	3.67
BIO13	Precipitation of wettest month	4	4.09
BIO14	Precipitation of driest month	15	2.97

Note: Variable importance was determined using Permutation Importance calculated from the Random Forest model for each of the 69 species. The ranks were derived from the per-species importance values (1st = most important, 6th = least important). † Mean Rank was computed across all 69 species, where a lower value indicates greater overall importance in determining species distribution. The results show that the thermal (BIO1) and dry-season precipitation (BIO14) factors are the primary drivers.

**Table 5:** Environmental variable combination patterns of species.

Variable Combination Pattern	Definition (Permutation-Importance Ranks)	Number of Species	Percentage (%)
Temperature-centered	All of top-3 in BIO1–BIO3 (temperature variables)	5	7.2
Precipitation-centered	All of top-3 in BIO12–BIO14 (precipitation variables)	2	2.9
Mixed (Temp. + Precip.)	Top-3 contains variables from both groups	62	89.9
Total		69	100

## Data Availability

The original contributions presented in this study are included in the article/supplementary material. Further inquiries can be directed to the corresponding author.

## Supplementary Materials

The following supporting information can be downloaded at: https://www.mdpi.com/article/10.3390/plants14243735/s1, Table S1: Legally protected endangered vascular plant species modeled in this study, with their national legal conservation status under the Korean Wildlife Protection and Management Act and Korean National Red List categories based on IUCN criteria. Table S2: Species-specific Random Forest (RF) model performance and climatic response characteristics for 69 endangered plant species in South Korea.

## Abbreviations

The following abbreviations are used in this manuscript:

AUC	Area under the receiver operating characteristic curve
ROC	Receiver operating characteristic
TSS	True Skill Statistic
VIF	Variance Inflation Factor
CMIP6	Coupled Model Intercomparison Project Phase 6
