# Supplementary material for: Mapping Endangered Plant Distributions, Species Richness, and Climate Refugia Under SSP Climate Scenarios in South Korea
Source: Plants (Basel). 2025 Dec 8;14(24):3735. doi: 10.3390/plants14243735 (PMC12736564; doi:10.3390/plants14243735)
Supplement: Supplementary file 1 [file plants-14-03735-s001.zip › supplymentary materials (table s1)_revision2.pdf]

Table S1. Legally protected endangered vascular plant species modelled in this study, with their national legal conservation status under the Korean Wildlife Protection and Management Act and Korean National Red List categories based on IUCN criteria.

No.	Scientific name	Legal conservation status (Endangered Wild Plant)	Korean National Red List category
1	*Euryale ferox*	Class II	VU
2	*Eleutherococcus senticosus*	Class II	VU
3	*Nymphaea tetragona* var. *minima*	Class II	CR
4	*Quercus gilva*	Class II	NT
5	*Glaux maritima* var. *obtusifolia*	Class II	NT
6	*Cypripedium japonicum*	Class I	EN
7	*Hemipilia cucullata*	Class II	EN
8	*Gastrochilus fuscopunctatus*	Class I	CR
9	*Trientalis europaea*	Class II	NT
10	*Drosera peltata* var. *nipponica*	Class II	NT
11	*Kirengeshoma koreana*	Class II	EN
12	*Phalaenopsis japonica*	Class I	RE
13	*Lilium dauricum*	Class II	EN
14	*Viola mirabilis*	Class II	EN
15	*Iris koreana*	Class II	VU
16	*Aster danyangensis*	Class II	EN

No.	Scientific name	Legal conservation status (Endangered Wild Plant)	Korean National Red List category
17	*Anagallidium dichotomum*	Class II	EN
18	*Cymbidium macrorhizon*	Class II	VU
19	*Cicuta virosa*	Class II	NT
20	*Euchresta japonica*	Class I	CR
21	*Ranunculus kadzusensis*	Class II	NT
22	*Lasianthus japonicus*	Class II	CR
23	*Ceratopteris thalictroides*	Class II	NT
24	*Aconitum coreanum*	Class II	VU
25	*Orobanche filicicola*	Class II	VU
26	*Odontochilus nakaianus*	Class II	EN
27	*Cypripedium macranthos*	Class II	VU
28	*Silene capitata*	Class II	VU
29	*Thrixspermum japonicum*	Class I	EN
30	*Viburnum burejaeticum*	Class II	NT
31	*Paeonia obovata*	Class II	EN
32	*Saururus chinensis*	Class II	EN
33	*Dendrobium moniliforme*	Class II	VU
34	*Viola raddeana*	Class II	CR

No.	Scientific name	Legal conservation status (Endangered Wild Plant)	Korean National Red List category
35	*Cotoneaster wilsonii*	Class II	EN
36	*Bupleurum latissimum*	Class II	EN
37	*Scrophularia takesimensis*	Class II	CR
38	*Aconitum austrokoreense*	Class II	NT
39	*Psilotum nudum*	Class II	EN
40	*Brasenia schreberi*	Class II	VU
41	*Diapensia lapponica* var. *obovata*	Class I	CR
42	*Pedicularis ishidoyana*	Class II	VU
43	*Thalictrum coreanum*	Class II	VU
44	*Viola websteri*	Class II	VU
45	*Cyrtosia septentrionalis*	Class II	NT
46	*Utricularia yakusimensis*	Class II	NT
47	*Dysophylla yatabeana*	Class II	EN
48	*Amsonia elliptica*	Class II	VU
49	*Lychnis wilfordii*	Class II	EN
50	*Mankyua chejuensis*	Class I	CR
51	*Menyanthes trifoliata*	Class II	NT
52	*Cymbidium lancifolium*	Class I	CR

No.	Scientific name	Legal conservation status (Endangered Wild Plant)	Korean National Red List category
53	*Sarcandra glabra*	Class II	EN
54	*Pelatantheria scolopendrifolia*	Class II	VU
55	*Lycoris chinensis* var. *sinuolata*	Class II	VU
56	*Oberonia japonica*	Class II	CR
57	*Halenia coreana*	Class II	VU
58	*Isoetes coreana*	Class II	NT
59	*Michelia compressa*	Class II	EN
60	*Metanarthecium luteo-viride*	Class II	VU
61	*Bulbophyllum drymoglossum*	Class II	EN
62	*Epilobium hirsutum*	Class II	NT
63	*Cypripedium guttatum*	Class I	CR
64	*Neofinetia falcata*	Class I	EN
65	*Pedicularis hallaisanensis*	Class II	EN
66	*Cymbidium kanran*	Class I	EN
67	*Habenaria radiata*	Class II	EN
68	*Bulbophyllum inconspicuum*	Class II	VU
69	*Arctous rubra*	Class II	EN

Notes:

Legal conservation status refers to the national designation of Endangered Wild Plants by the Ministry of

Environment (Korea):

Class I = species at very high risk of extinction in the wild;

Class II = species at high risk of extinction in the wild.

Korean National Red List categories follow IUCN criteria: RE = Regionally Extinct; CR = Critically Endangered; EN = Endangered; VU = Vulnerable; NT = Near Threatened.
