# Supplementary material for: Mapping Endangered Plant Distributions, Species Richness, and Climate Refugia Under SSP Climate Scenarios in South Korea
Source: Plants (Basel). 2025 Dec 8;14(24):3735. doi: 10.3390/plants14243735 (PMC12736564; doi:10.3390/plants14243735)
Supplement: Supplementary file 1 [file plants-14-03735-s001.zip › supplymentary materials (table s2)_revision2.pdf]

Table S2. Species-specific Random Forest (RF) model performance and climatic response characteristics for 69 endangered plant species in South Korea.

No.	Scientific Name	AUC	Performance Class	Primary Driver (BIO Code)	Primary Driver Group	Combination Pattern
1	*Euryale ferox*	0.892	Very Good	BIO1	Temperature	Mixed
2	*Eleutherococcus senti cosus*	0.933	Excellent	BIO1	Temperature	Mixed
3	*Nymphaea tetragona* var. *minima*	0.718	Good	BIO14	Precipitation	Mixed
4	*Quercus gilva*	0.995	Near-perfect	BIO1	Temperature	Mixed
5	*Glaux maritima* var. *obtusifolia*	0.700	Good	BIO13	Precipitation	Mixed
6	*Cypripedium japonicum*	0.850	Very Good	BIO1	Temperature	Mixed
7	*Hemipilia cucullata*	0.882	Very Good	BIO1	Temperature	Mixed
8	*Gastrochilus fuscopunctatus*	0.942	Excellent	BIO12	Precipitation	Mixed
9	*Trientalis europaea* subsp. *arctica*	0.939	Excellent	BIO1	Temperature	Mixed
10	*Drosera peltata* var. *nipponica*	0.970	Excellent	BIO1	Temperature	Mixed
11	*Kirengeshoma koreana*	0.981	Near-perfect	BIO12	Precipitation	Mixed
12	*Phalaenopsis japonica*	0.990	Near-perfect	BIO1	Temperature	Mixed
13	*Lilium dauricum*	0.901	Excellent	BIO1	Temperature	Mixed
14	*Viola mirabilis*	0.957	Excellent	BIO1	Temperature	Temperature-centered
15	*Iris koreana*	0.935	Excellent	BIO14	Precipitation	Mixed
16	*Aster danyangensis*	0.882	Very Good	BIO2	Temperature	Mixed
17	*Anagallidium dichotomum*	0.961	Excellent	BIO1	Temperature	Mixed
18	*Cymbidium macrorhizon*	0.919	Excellent	BIO12	Precipitation	Mixed
19	*Cicuta virosa*	0.925	Excellent	BIO1	Temperature	Temperature-centered
20	*Euchresta japonica*	0.997	Near-perfect	BIO12	Precipitation	Mixed
21	*Ranunculus*	0.963	Excellent	BIO14	Precipitation	Mixed

	kazusensis					
22	*Lasianthus japonicus*	0.998	Near-perfect	BIO1	Temperature	Mixed
23	*Ceratopteris thalictroides*	0.938	Excellent	BIO1	Temperature	Mixed
24	*Aconitum coreanum*	0.948	Excellent	BIO14	Precipitation	Mixed
25	*Orobanche filicicola*	0.767	Good	BIO14	Precipitation	Mixed
26	*Odontochilus nakaianus*	0.986	Near-perfect	BIO14	Precipitation	Mixed
27	*Cypripedium macranthos*	0.877	Very Good	BIO1	Temperature	Mixed
28	*Silene capitata*	0.890	Very Good	BIO12	Precipitation	Mixed
29	*Thrixspermum japonicum*	0.999	Near-perfect	BIO14	Precipitation	Precipitation-centered
30	*Viburnum burejaeticum*	0.887	Very Good	BIO2	Temperature	Mixed
31	*Paeonia obovata*	0.941	Excellent	BIO1	Temperature	Mixed
32	*Saururus chinensis*	0.969	Excellent	BIO2	Temperature	Mixed
33	*Dendrobium moniliforme*	0.856	Very Good	BIO2	Temperature	Mixed
34	*Viola raddeana*	0.462	Random	BIO1	Temperature	Mixed
35	*Cotoneaster wilsonii*	0.999	Near-perfect	BIO14	Precipitation	Mixed
36	*Scrophularia takesimensis*	1.000	Perfect	BIO12	Precipitation	Mixed
37	*Bupleurum latissimum*	1.000	Perfect	BIO12	Precipitation	Mixed
38	*Aconitum austrokoreense*	0.864	Very Good	BIO14	Precipitation	Mixed
39	*Psilotum nudum*	0.954	Excellent	BIO1	Temperature	Mixed
40	*Brasenia schreberi*	0.899	Very Good	BIO14	Precipitation	Mixed
41	*Diapensia lapponica* var. *obovata*	1.000	Perfect	BIO14	Precipitation	Precipitation-centered
42	*Pedicularis ishidoyana*	0.899	Very Good	BIO1	Temperature	Mixed
43	*Thalictrum coreanum*	0.919	Excellent	BIO1	Temperature	Mixed
44	*Viola websteri*	0.857	Very Good	BIO1	Temperature	Mixed
45	*Cyrtosia septentrionalis*	0.795	Very Good	BIO14	Precipitation	Mixed
46	*Utricularia yakusimensis*	0.856	Very Good	BIO14	Precipitation	Mixed
47	*Dysophylla yatabeana*	0.843	Very Good	BIO1	Temperature	Mixed

48	*Amsonia elliptica*	0.999	Near-perfect	BIO3	Temperature	Temperature-centered
49	*Lychnis wilfordii*	0.997	Near-perfect	BIO1	Temperature	Mixed
50	*Mankyua chejuensis*	0.998	Near-perfect	BIO12	Precipitation	Mixed
51	*Menyanthes trifoliata*	0.838	Very Good	BIO2	Temperature	Temperature-centered
52	*Cymbidium lancifolium*	0.998	Near-perfect	BIO3	Temperature	Mixed
53	*Sarcandra glabra*	0.999	Near-perfect	BIO3	Temperature	Mixed
54	*Pelatantheria scolopendrifolia*	0.936	Excellent	BIO1	Temperature	Mixed
55	*Lycoris chinensis* var. *sinuolata*	0.982	Near-perfect	BIO14	Precipitation	Mixed
56	*Oberonia japonica*	0.992	Near-perfect	BIO14	Precipitation	Mixed
57	*Halenia corniculata*	0.931	Excellent	BIO1	Temperature	Mixed
58	*Isoetes coreana*	0.775	Good	BIO2	Temperature	Mixed
59	*Michelia compressa*	0.996	Near-perfect	BIO3	Temperature	Mixed
60	*Metanarthecium luteo-viride*	0.963	Excellent	BIO12	Precipitation	Mixed
61	*Bulbophyllum drymoglossum*	0.932	Excellent	BIO14	Precipitation	Mixed
62	*Epilobium hirsutum*	0.821	Very Good	BIO1	Temperature	Mixed
63	*Cypripedium guttatum*	0.665	Fair	BIO1	Temperature	Mixed
64	*Neofinetia falcata*	0.989	Excellent	BIO2	Temperature	Mixed
65	*Pedicularis hallaisanensis*	0.909	Excellent	BIO1	Temperature	Mixed
66	*Cymbidium kanran*	0.977	Near-perfect	BIO12	Precipitation	Mixed
67	*Habenaria radiata*	0.982	Near-perfect	BIO3	Temperature	Mixed
68	*Bulbophyllum inconspicuum*	0.993	Near-perfect	BIO14	Precipitation	Mixed
69	*Arctous rubra*	0.573	Poor	BIO1	Temperature	Temperature-centered

Note: AUC (Area Under the ROC Curve) values were obtained from repeated 5-fold cross-validation (10 repetitions). Performance classes follow standard SDM evaluation criteria defined in Table 3: Perfect (1.000), Near-perfect (0.990–0.999), Excellent (0.900–0.989), Very Good (0.800–0.899), Good (0.700–0.799), Fair (0.600–0.699), Poor (0.500–0.599), Random (<0.500). Primary Driver indicates the bioclimatic variable with the highest permutation importance for each species. Primary Driver Group categorizes variables as temperature-related (BIO1–BIO3) or precipitation-related (BIO12–BIO14). Combination Pattern classifies species based on their top three most important variables: Temperature-centered (all top-3 variables are

BIO1–BIO3), Precipitation-centered (all top-3 variables are BIO12–BIO14), or Mixed (combination of both temperature and precipitation variables).
